# Characterization of native *Escherichia coli* populations from bovine vagina of healthy heifers and cows with postpartum uterine disease

**DOI:** 10.1371/journal.pone.0228294

**Published:** 2020-06-01

**Authors:** Candelaria Gonzalez Moreno, Andrea Torres Luque, Rubén Oliszewski, Ramiro J. Rosa, María C. Otero

**Affiliations:** 1 Instituto Superior de Investigaciones Biológicas (INSIBIO), Consejo Nacional de Investigaciones Científicas y Técnicas (CONICET), San Miguel de Tucumán, Tucumán, Argentina; 2 Instituto de Biología "Dr. Francisco D. Barbieri", Facultad de Bioquímica, Química y Farmacia, Universidad Nacional de Tucumán (UNT), San Miguel de Tucumán, Tucumán, Argentina; 3 Facultad de Agronomía y Zootecnia, Universidad Nacional de Tucumán, El Manantial, Tucumán, Argentina; 4 Laboratorio de Calidad de Lácteos (LaCaLac), Facultad de Agronomía y Zootecnia, Universidad Nacional de Tucumán (UNT), Consejo Nacional de Investigaciones Científicas y Técnicas (CONICET), El Manantial, Tucumán, Argentina; Ross University School of Veterinary Medicine, SAINT KITTS AND NEVIS

## Abstract

Even though *Escherichia coli* are common bacteria of the bovine vaginal microbiota, they represent an important pathogen that causes diseases in the reproductive tract and subfertility. However, the actual endometrial virulence profile of *E*. *coli* is poorly understood. The present study aims to characterize the phylogenetic structure and virulence potential of native vaginal populations of *E*. *coli* from healthy heifers (H), and cows with postpartum uterine diseases (PUD), such as metritis/endometritis (MT) or repeat breeder cows (RB). To this end, the virulence repertoire of 97 *E*. *coli* isolates was genotypically and phenotypically assessed. Most of them were assigned to phylogenetic group A (74%), followed by B1 (17%) and D (9%); RB strains were significantly (*p* < 0.05) more represented by B1. Seven of the 15 evaluated virulence genes (VFG) were detected and the most prevalent were *fimH* (87%), *agn43* (41%) and *csgA* (35%); while *traT* (27%), *fyuA* (11%), *hlyA* (5%) and *kpsMT II* (5%) were observed in a lower proportion. Particularly, *fyuA* was significantly higher (*p* < 0.05) in MT cows whereas *csgA* showed the same behavior in PUD animals (*p* < 0.05). When comparing H and PUD strains, these last ones were associated to positive expression of biofilm, fimbriae curli/cellulose and motility; yet RB strains did not show motility. Vaginal B1 *E*. *coli* populations, that possess VFG (*fyuA* and *csgA*) as well as the expression of motility, curli fimbriae/cellulose and biofilm, may represent risk factors for endometrial disorders; specifically, those that also, have *kpsMT II* may have a pathogenic potential for causing the RB syndrome. Future research focusing on the detection of these strains in the vaginal microbiota of cows with postpartum uterine diseases should be done since the control of their presence in vagina could reduce the risk that they access the uterus during the postpartum period.

## Introduction

Ascending infection from the bovine vagina after parturition frequently leads to disease of the upper female genital tract [1,2]. Postpartum uterine disease (puerperal metritis, clinical and subclinical endometritis) have a significant impact in dairy farms productivity because they perturb reproductive performance and contribute to cow discomfort, reduce milk production, increase treatment costs, antibiotic therapies and culling rates [3–7]. Furthermore, postpartum metritis and endometritis may lead to the Repeat Breeding Syndrome (RBS) [8]. A repeat breeder (RB) is usually defined as a cow with apparently healthy genitalia that cannot conceive after three or more artificial inseminations or natural mating but exhibits normal estrous cycles [9]. This syndrome is one of the most important reproductive disorders in dairy cows that have the subclinical endometritis as one of its main causes [8]. Bacterial infection can modify the uterine environment producing inflammation, denudation of the mucosa and changes in its secretion with subsequent embryonic death [8], which results in an increase of days open and number of services per conception, as well as in early culling [5, 10, 11].

*Escherichia coli* is one of the most relevant bacteria implicated in the establishment of postpartum uterine diseases; also increases the uterus susceptibility to subsequent infections by *Arcanobacterium pyogenes* and other bacterial species [12–15]. It has been reported that *E*. *coli* colonization of the upper reproductive tract is associated with severe damage to the endometrium and disruption of the ovarian cyclic activity followed by infertility [16–19]. Moreover, *E*. *coli* lipopolysaccharide (LPS) disrupts endocrine function by decreasing peripheral plasma concentrations of estradiol and progesterone, as well as suppressing GnRH and LH secretions by the hypothalamus and pituitary gland, respectively [18]. It is also responsible for switching prostaglandin uterine secretion to predominantly prostaglandin E2, which prolongs the calving interval [18]. It is important to keep in mind that, while *E*. *coli* is recognized as a bovine metritic pathogen, this bacterium has also been recovered from the vagina of heifers from weaning to breeding; likewise, it was isolated from the postpartum uterus of cows that did not develop clinical disease [20–22]. Nevertheless, most non-culturable efforts to study vaginal and uterine microbial communities of cows had reported that *E*. *coli* has turned out to be very rare [23–25]; meaning that *E*. *coli* is in a low relative abundance in the reproductive tract. These contradictory results may be due to the diversity of *E*. *coli* strains and the existence of virulent clones [25]. In Fact, Silva et al. [14] reported genomic diversity of uterine *E*. *coli* isolates from dairy cows with metritis, and Sheldon et al. [2] proposed that there are endometrial pathogenic strains that are more invasive and adherent to endometrial stromal cells than other strains of *E*. *coli*. Thus, in order to define a specific profile for endometrial pathogenicity, several authors have partially characterized *E*. *coli* isolated from the postpartum bovine uterus nevertheless; no consensus has been reached on this issue [2, 4, 14, 15, 26]. These studies evaluated several virulence factor genes (VFG) with divisive results; therefore, the association between a pathogenicity profile of *E*. *coli* and the establishment of puerperal uterine disease is still unclear. Some of these studies [2,14] also evaluated the relationship between the *E*. *coli* phylogeny and virulence profile. Thus, Sheldon et al [2] concluded that isolates from the uterus of cows with metritis belonged predominantly to B1 and A, while those from the uterus of healthy cows were distributed more equally among the groups A, B1 and D. However, genomic characteristics of bovine vaginal *E*. *coli* and their association with virulence factors and uterine diseases have not been assessed yet.

Therefore, the aim of this study was to characterize native populations of *E*. *coli* from vagina of either healthy heifers or cows with postpartum uterine diseases (metritic, endometritic or RB cows), through genomic characterization including the evaluation of genetic diversity, phylogeny, VFG profiles (presence of genes by PCR) and assessing phenotypic features, such as the expression of colonization-associated properties (expression of type 1 and P fimbriae, biofilm formation, curli fimbriae and cellulose production).

## Materials and methods

### Farm selection and sample collection

All procedures were conducted under the Argentinean Animal Welfare Legislation, Law N°14.346, SENASA-R70/2001 with the approval of the Institutional Committee for the Care and Use of Laboratory Animals of the National University of Tucumán (CICUAL–UNT, Research Protocol N° 030/2019). The animal management procedures included in the experimental protocol (intra-vaginal treatments, hormonal injections and vaginal sampling) were conducted by trained veterinarians and are part of routine veterinary practices for these animals. Data were monthly collected from Holstein healthy heifers (n = 48) as well as cows with puerperal uterine diseases (n = 49) from three dairy farms (El Potrerillo, SA Azucarera Justiniano Frías Silva and San José) near Trancas city, Tucumán Province (North West of Argentina). These farms were selected because of their long working relationship with the Veterinary College at the National University of Tucuman. All animals were maintained under standard conditions and examined as described during a one-year period [27]. Heifers selection was based on the following criteria: 18-month-old (± 2 months) nulliparous healthy animals that have not been previously inseminated or used for natural breeding and were deemed suitable for reproduction through transrectal palpation to confirm reproductive maturity. On the other hand, cows with puerperal uterine diseases were included on the experiment: cows with up to 60 days postpartum metritis or endometritis (MT, n = 13) and repeat breeder cows (RB) unable to conceive after three or more artificial inseminations or natural mating (n = 36).

Uterine disease was evaluated as described previously with animals categorized as having metritis if they had fetid, watery, red-brown uterine discharge, associated with fever (rectal temperature > 39.5°C), and systemic signs of illness (dullness, reduced appetite, and milk production) within the first 21 days in milking (DIM) [3], whereas clinical endometritis was defined as the presences of mucopurulent or purulent uterine discharge 21–60 DIM [4, 28].

In order to take the samples from the vaginal fornix, animals were restrained in the cattle crush. Briefly, after cleaning the perineum and vulvar areas, a steel vaginoscope was placed into the posterior area of the vagina using antiseptic-free lubricant gel (0.8% w/v carbopol, 1% v/v triethanolamine, 10% v/v glycerol; Sigma-Aldrich) and a long-handled sterile cytobrush was used to scrape the cranial vaginal wall. Then, the cytobrush was placed in a sterile tube containing 1 mL phosphate buffered saline solution (PBS, pH 7.0) and kept refrigerated (4°C) until processing.

### Isolation and characterization of native *E*. *coli*

Vaginal swabs were taken refrigerated to the laboratory and cultured in MacConkey agar (Britania Laboratories, Argentina) at 37°C for 24 h followed by a random selection of 3–4 lactose positive colonies from each sample for further bacterial identification. *E*. *coli* isolates were identified by both, standard biochemical (indole production, lactose and citrate utilization, methyl red and Voges-Proskauer reaction) [29] and molecular biology techniques (*uidA* detection) [30, 31]; primers sequences and annealing temperature are indicated in Table 1. All *E*. *coli* isolates were stored on 20% glycerol supplemented BHI medium at -20°C. *E*. *coli* EDL933 and B41 were used as control strains.

### Genomic DNA extraction

Isolates of *E*. *coli* and reference strains were grown on BHI agar (Britania Laboratories, Argentina) at 37°C for 16 h. DNA was extracted as follows: bacterial cells were washed with 200 μL of sodium chloride buffer-Tris-EDTA (STE) (150 mM NaCl, 50 mM Tris, 50 mM Na_2_-EDTA). Afterwards, they were suspended in 200 μL of PBS followed by the addition of 10 μL of Proteinase K (600 mUA/mL) and lysis buffer (Buffer AL Qiagen, Germany). After incubation (56°C, 10 min), 140 μL of 5 M NaCl and chloroform (25: 1) were added, subsequently DNA precipitation with isopropanol was performed [32]. Finally, DNA samples were washed with 70° ethanol, centrifuged, left to dry and then suspended in nuclease-free water. DNA concentration and purity were assessed by optical density, also the integrity was evaluated through a 0.8% agarose gel electrophoresis.

### Bacterial genetic diversity analysis

Enterobacterial repetitive intergenic consensus PCR (ERIC-PCR) was conducted using the primer ERIC2 (5 'AAGTAAGTGACTGGGGTGAGCG 3') to evaluate the genetic diversity of *E*. *coli* strains; the methodology employed was previously described by Versalovic et al. [33] with minor modifications. A negative control (no DNA) was included in each PCR assay. The reaction mixture (25 μL) contained 7 μL of sterile distilled water, 10 μM of the ERIC2 primer, 50 ng of DNA template, 2.5 μM of each dNTP, 2 U of the Taq DNA polymerase (Promega, USA), 1x Taq buffer and 1.5 mM of MgCl_2_.

PCR conditions were: 95°C for 7 min; 35 cycles of 94°C for 1 min, 45°C for 1 min and 72°C for 8 min; and a final extension of 72°C for 15 min. PCR products were resolved by electrophoresis on a 1.5% agarose gel (w/v) containing Diamond^™^ Nucleic Acid Dye (Promega, EE.UU.) at 70 V for 6 h. Also, band sizes were determined by comparison with a standard DNA ladder (50 bp DNA Ladder GeneRuler, Thermo Fisher Scientific, USA). DNA fingerprints were analyzed in Bionumerics version 7.6 from Applied Maths (Sint-Martens-Latem, Belgium). The similarity degree among the genetic fingerprints was calculated using the Pearson Correlation Coefficient and clustering was based on the unweighted pair group method using arithmetic averages (UPGMA).

### Phylogenetic grouping

Phylogenetic grouping of *E*. *coli* isolates was done based on criteria previously proposed [34] which allows the discrimination of four phylogenetic groups: A, B1, B2, and D by applying a multiplex PCR targeting the genes *chuA* and *yjaA*, and the DNA fragment TspE4-C2. PCR reaction mixture (25 μL) contained 12.5 of μL GoTaq Green Master Mix (2X) (Promega, USA), 1 μL of template and a concentration of 0.08 μM of each primer (Table 1). Amplification conditions included: an initial denaturation of the DNA for 5 min at 95°C, followed by 30 cycles (denaturation for 5 s at 95°C, annealing for 10 s at 59°C and extension for 30 s at 72°C) and a single final extension cycle for 5 min at 72°C. Amplification products were separated by electrophoresis through a 2% (w/v) agarose gel containing GelRed (Biotium, USA). A 50 bp DNA Ladder Gene Ruler (Thermo Fisher Scientific, USA) was used as a molecular-weight marker to recognize the expected product sizes. *E*. *coli* 536 and verotoxin-producing *E*. *coli* O157:H7 (EDL933) were used as positive controls for phylogenetic groups B2 and D, respectively.

**Table 1:** Primers used in PCR protocols of *E*. *coli* identification and phylogenetic/virulence characterization.

Gene	Primer secuence (5´-3´)	T° annealing	Size of products	Reference
*uidA*	TGTTACGTCCTGTAGAAAGCCC AAAACTGCCTGGCACAGCAATT	58°C	154 bp	[35]
*chuA*	GACGAACCA ACGGTCAGGAT TGCCGCCAGTACC AAAGACA	58°C	279 bp	[34]
*yjaA*	TGAAGTGTCAGGAGACGCTG ATGGAGAATGCGTTCCTCAAC	58°C	211 bp	[34]
TspE4C2*	GAGTAATGTCGGGGCATTCA CGCGCCAACAAAGTATTACG	58°C	152 bp	[34]
*fimH*	TGCAGAACGGATAAGCCGTGG GCAGTCACCTGCCCTCCGGTA	63°C	508 bp	[36]
*papC*	GACGGCTGTACTGCAGGGTGTGGCG ATATCCTTTCTGCAGGGATGCAATA	50°C	380 bp	[36]
*sfa/focDE*	CTCCGGAGAACTGGGTGCATCTTAC CGGAGGAGTAATTACAAACCTGGCA	63°C	410 bp	[36]
*afa/draBC*	GGCAGAGGGCCGGCAACAGGC CCCGTAACGCGCCAGCATCTC	63°C	559 bp	[36]
*hlyA*	AACAAGGATAAGCACTGTTCTGGCT ACCATATAAGCGGTCATTCCCGTCA	63°C	1177 bp	[36]
*fyuA*	TGATTAACCCCGCGACGGGAA CGCAGTAGGCACGATGTTGTA	63°C	880 bp	[36]
*iutA*	GGCTGGACATCATGGGAACTGG CGTCGGGAACGGGTAGAATCG	68°C	301 bp	[36]
*kpsMT II*	GCGCATTTGCTGATACTGTTG CATCCAGACGATAAGCATGAGCA	63°C	272 bp	[36]
*traT*	GGTGTGGTGCGATGAGCACAG CACGGTTCAGCCATCCCTGAG	63°C	290 bp	[36]
*agn43*	CTGGAAACCGGTCTGCCCTT CCTGAACGCCCAGGGTGATA	58°C	433 bp	[37]
*csgA*	ACTCTGACTTGACTATTACC AGATGCAGTCTGGTCAAC	55°C	200 bp	[38]
*Eae*	GGAACGGCAGAGGTTAATCTGCAG GGCGCTCATCATAGTCTTTC	55°C	775 bp	[39]
Gene encoding F41	GCATCAGCGGCAGTATCT GTCCCTAGCTCAGTATTATCACCT	50°C	380 bp	[40]
Gene encoding F5 (K99)	TATTATCTTAGGTGGTATGG GGTATCCTTTAGCAGCAGTATTTC	50°C	314 bp	[40]
Gene encoding *STa*	GCTAATGTTGGCAATTTTTATTTCTGTA AGGATTACAACAAAGTTCACAGCAGTAA	50°C	190 bp	[40]

*anonymous fragment

### Detection of Virulence Factors Genes (VFG)

Each strain was screened by conventional PCR analysis for the presence of 15 VFG. Some of them were related to ExPEC while others were associated to diarrheagenic *E*. *coli* pathotypes (ETEC and EPEC): *fimH* (type 1 fimbriae), *papC* (P fimbriae), *sfa/focDE* (S fimbriae), *csgA* (curli fimbriae), *afa*/draBC (Dr-binding adhesin), *fyuA* (yersiniabactin), *iutA* (aerobactin), *hlyA* (hemolysin A), *traT* (serum resistance), *kpsMT II* (protectin), *agn43* (biofilm), *eae* (intimin), F41 (fimbriae), F5 (fimbriae) and *sta* (heat-stable enterotoxin). All reactions were performed in a 25 μL volume using primers detailed in Table 1 [2, 4, 14]. Briefly, the reaction mixture contained 5 μL of buffer-MgCl_2_ (10x), 1 μL of each primer (10 μM) (Table 1), 2 μL of dNTP mix (di-deoxynucleotides, 2.5 mM), 0.15 μL of Taq (5 U/μL) (Promega, USA), 13.85 μL of nuclease free water and 2 μL of DNA template. Amplification products were separated by electrophoresis through a 1% (w/v) agarose gel, stained with GelRed (Biotium, USA); those amplicons that had the expected molecular size were considered to be positives. The *E*. *coli* strains used as positive controls were: BEN2908 (APEC *fimH*; *iutA*; *ibeA*), BEN312 (ExPEC *afa*; *papC*), EDL933 (STEC *eae*; *agn43*), B41 (ETEC F5; F41; *sta*), 536 (UPEC *sfaS*; *fyuA*) and a clinical isolate *E*. *coli* HdN3 (UPEC *hlyA*; *cnf1*; *traT*; *kpsMT II*).

### Hemolysin production

Alpha and/or beta hemolysin detection was performed by the analysis of the hemolytic zone observed after overnight growth at 37°C on sheep blood (5%) agar [41].

### Motility assay

The motility assay was modified from the method described by Kao et al. [42]. In brief, several grown colonies were stabbed on Luria Bertani soft agar plates (LB 0.3% agar) and incubated at 37°C for 8 h. Motility was assessed by the diameter of swimming halos, from the inoculated center toward the periphery of the plate. Motility of each isolate was measured as the mean of three independent experiments.

### Type 1 fimbriae and P fimbriae detection

Bacterial suspensions with 10^12^ CFU/mL (OD_540 nm_ = 1.2, in PBS) were prepared from over-night cultures obtained in LB agar from a single bacterial colony. Type 1 fimbriae expression was assayed by the bacterial ability to agglutinate yeast cells (*Saccharomyces cerevisiae*) as previously described [43]; thus, over-night (37°C) yeast cells suspensions grown in Sabouraud agar (Britania Laboratories, Argentina), were washed and suspended in PBS (OD_540 nm_ = 1.2). Fifty microlites of the two cellular suspensions were mixed on a glass slide for five minutes and the visualization of clusters was considered positive. Later, in those isolates proved positive, agglutination was assayed again in presence of D-mannose (1%). The presence of P fimbriae was assayed as previously described [29] with some small modifications. Briefly, human type A red blood cells were washed twice with PBS and suspended (5%); a volume of this suspension (50 μl) was mixed on a glass slide with an equal volume of the bacterial suspension (OD_540 nm_ = 1.2); after five minutes, hemagglutination was evaluated. In order to discard hemagglutination by type 1 fimbriae, the bacterial suspensions, previously treated with 1% D-mannose (30 minutes), were again tested. *Escherichia coli* ATCC 25922 and BEN312 were used as negative and positive controls for the mannose sensitive hemagglutination assay, respectively.

### Cellulose and/or curli fimbriae expression and biofilm formation

Bacterial suspensions (OD_540 nm_ = 1.0, in PBS) were prepared from an overnight bacterial culture (LB agar without NaCl, 37°C). Curli fimbriae expression was evaluated as previously described [44] from cultures obtained in LB broth (without NaCl) and incubated for 3 h at 30°C under shaking conditions. Then, 5 μL were spotted onto LB agar (without NaCl) supplemented with Congo Red (CR) (40 μg/mL) and Bromophenol Blue (20 μg/mL); after incubation (48 h, 30°C), the color and morphology of the colonies were evaluated. Biofilm formation on abiotic surfaces (polystyrene and glass) was assessed using previously described methods [45, 46]; thus, tubes containing 3 mL of LB (without NaCl) were inoculated with 30 μL of the bacterial suspensions and incubated for 48 h at 30°C without shaking. At the same time, aliquots (200 μl) were extracted from them and added to polystyrene microplate and incubated under the same experimental conditions. After the incubation period, the medium was gently aspirated from both, glass tubes and wells, washed twice with PBS and air-dried. Biofilms were stained with 0.1% (w/v) crystal violet solution for 30 min, then washed with distilled water and air-dried. The colorant was extracted with ethanol 96% (15 min) and OD_590 nm_ measurements were performed in aliquots from each glass tube and well. A known strain of *E*. *coli* repeatedly giving positive was taken as control for the assay.

### Statistical analyses

The data processing was carried out using three software: MINITAB (15 version), InfoStat (2015p version) and JMP (Pro 13 version). The tests were carried out in duplicate or triplicate and the data were evaluated statistically by a variance analysis; in those cases where the residuals showed a normal distribution a post test for multiple comparisons was performed; when it was not possible, a nonparametric variance analysis was applied (JMP software). Categorical data were evaluated through a contingency table (Fisher exact test) using InfoStat, and to express the relationship between the reproductive clinical condition (H, MT, RB) and virulence properties expression, a multivariate correspondence analysis was performed. Also, the ANOM test using MINITAB was applied to compare the score values for each animal group.

## Results

### *E*. *coli* from healthy heifers have a higher genetic diversity

**Fig 1:**
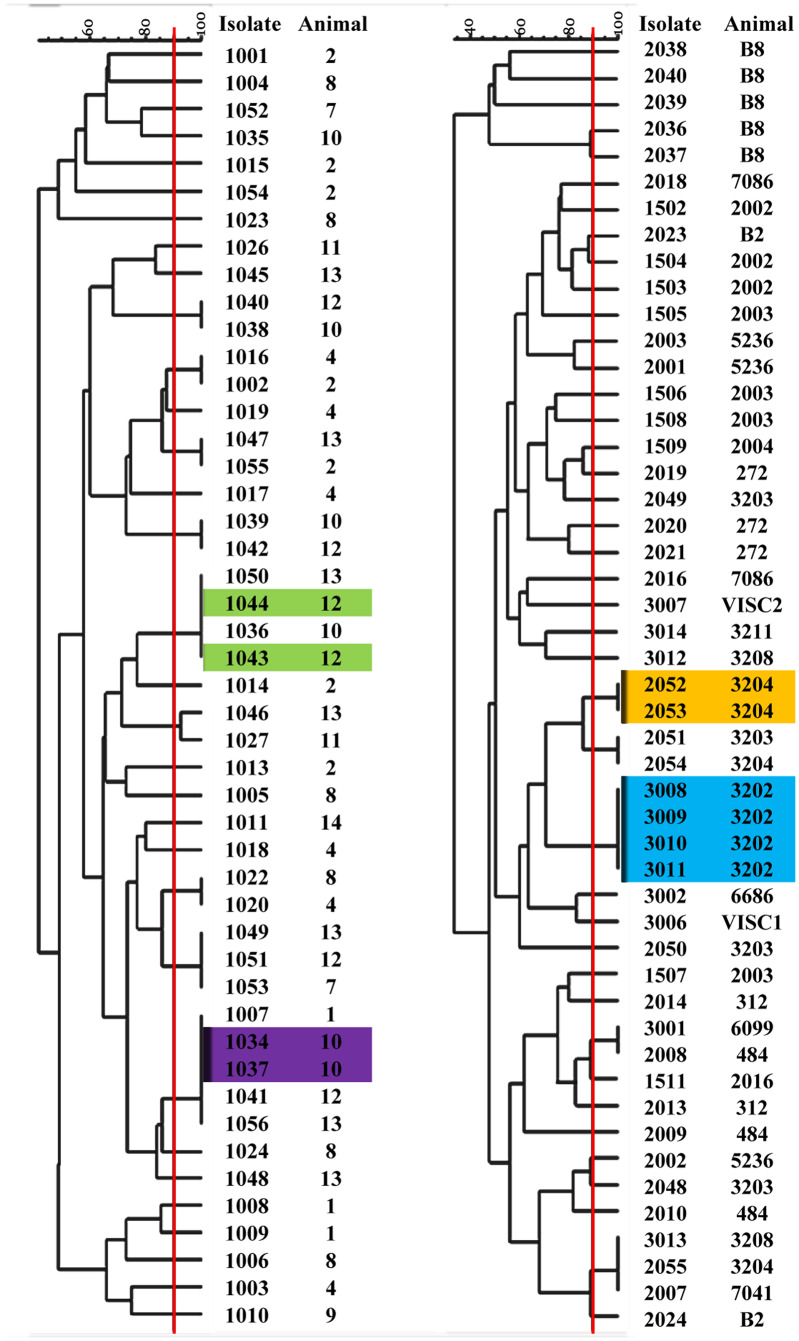
Dendrograms generated from fingerprints (ERIC-PCR) of bovine vaginal *E*. *coli*. The degree of similarity (%) between fingerprints is given at the top by the Pearson coefficient. The cut-off level ≥ 90% (red line) defined common clonal origin. Reference of isolates and animal origin are indicated on the right. Colored boxes indicate isolates from the same animal that presented patterns of similarity ≥ 90% have a common clonal origin. H: healthy heifers, PUD: cows with postpartum uterine diseases.

The fingerprinting profiles were analyzed according to the reproductive condition of the sampled animals in two different groups: healthy heifers (H) and cows with postpartum uterine diseases (PUD), which included metritis/endometritis and the repeat breeder syndrome (MT and RB) as indicated in Fig 1. The genetic similarity among *E*. *coli* from each animal group was 42–100% and 35–100%, for H and PUD, respectively. It was assumed that isolates from the same animal that presented patterns of similarity ≥ 90% have a common clonal origin. This result was only observed in four and six cases from H and PUD, respectively.

When analyzing the different patterns corresponding to a single animal, we observed that, in the H group there were up to seven different clonal profiles per animal; also 6/11 heifers exhibited more than five different profiles. Regarding PUD, a smaller number of patterns per animal was observed; where only one animal presented five different profiles; however, none of them showed a greater amount; also, 13 out of the 21 cows had only one or two different patterns (Fig 1, S1 Table).

### *E*. *coli* from phylogenetic group B1 were associated with repeat breeder (RB) cows

**Fig 2:**
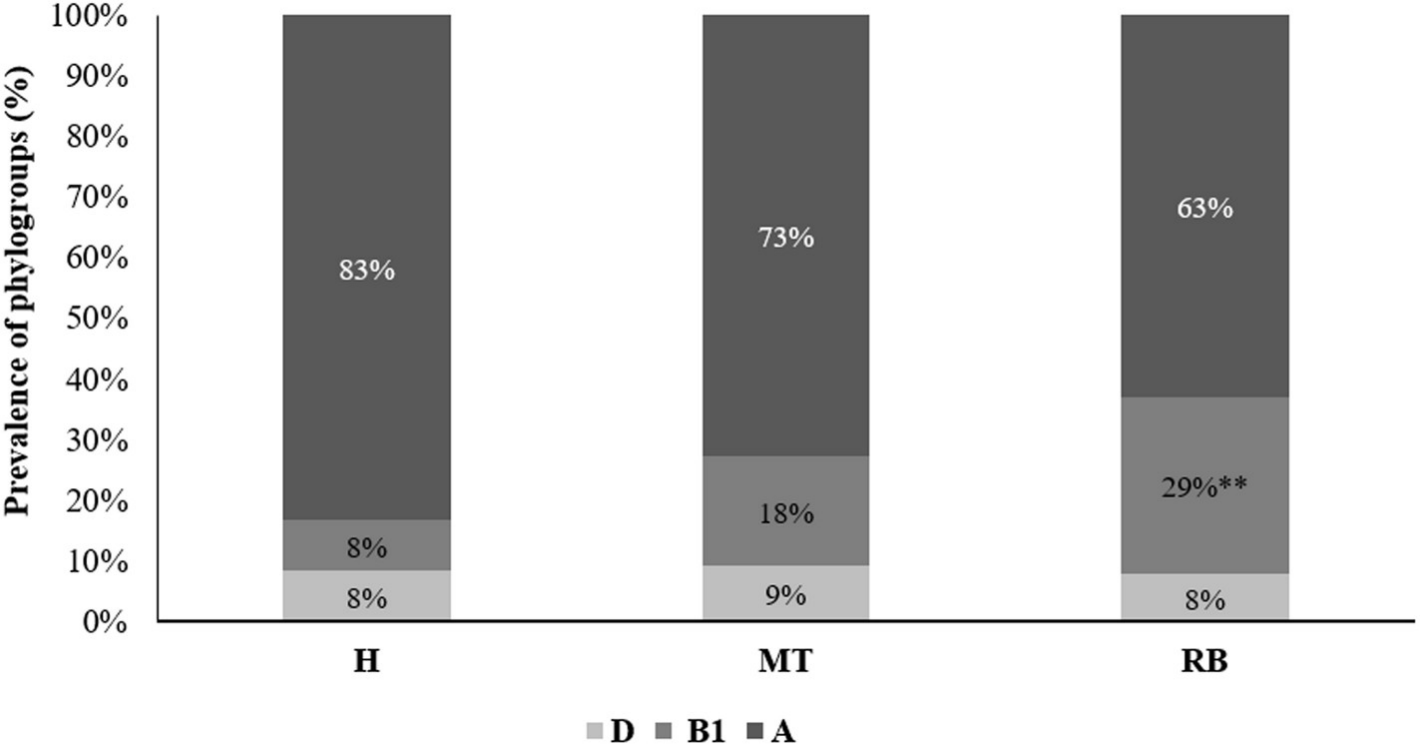
Phylogenetic classification of vaginal *E*. *coli* isolated from healthy heifers (H), cows with metritis or endometritis (MT) and repeat breeders (RB). **: indicate significant differences (*p* < 0.05 Fisher's exact test).

Bovine vaginal *E*. *coli* isolates were assigned to phylogenetic group A (74%), B1 (17%) or D (9%); however, none of them was classified as B2. The prevalence of group A was significantly higher (*p* < 0.05, Fisher's exact test) than B1 and D; and the general trend A > B1 > D was observed in the three animal groups. However, the prevalence of group D was mostly the same in the three groups and a trend of increasing prevalence of group B1 was observed from healthy to RB groups (*p* < 0.05, Fisher's exact test) (Fig 2).

### *E*. *coli* from cows with PUD possessed few genes commonly associated with extraintestinal pathogenicity

**Fig 3:**
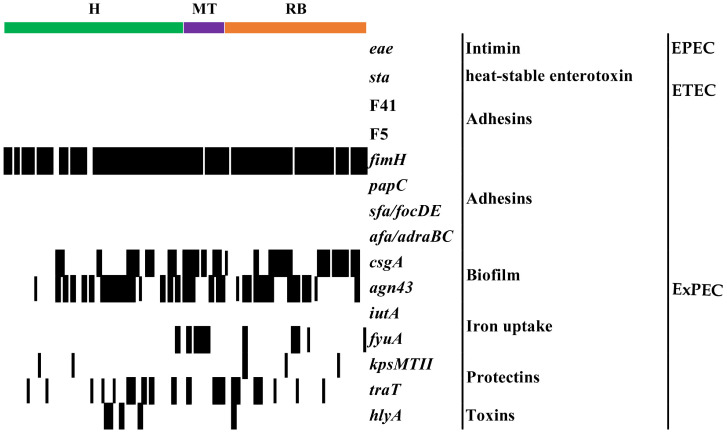
Fifteen VFG were evaluated in vaginal *E*. *coli* from healthy heifers (H), cows with metritis or endometritis (MT) and repeat breeders (RB). The matrix on the left indicates positive or negative detection; **: indicate significant differences (*p* < 0.05, Kruskal Wallis test).

To evaluate *E*. *coli* potential ability for uterine colonization and subsequent establishment of postpartum disease, 15 virulence genes associated with motility, adhesion and invasion, were examined (Fig 3). Only seven VFG were found among the 97 isolates: *fimH*, *csgA*, *agn43*, *fyuA*, *kpsMT II*, *traT* and *hlyA* **(**S2A–S2E Fig). However, none of the VFG associated with intestinal pathogenic *E*. *coli* (EPEC and ETEC) were detected. The most frequent VFG was *fimH* (87%), followed by *agn43* (41%) and *csgA* (35%); which code for factors associated with bacterial adhesion, curli fimbriae production and biofilm formation. Other VFG such as *traT* (27%), *fyuA* (11%), *hlyA* (5%) and *kpsMT II* (5%) were observed in a lower proportion (Fig 3). The differential analysis between the animal groups showed that the frequency of *fyuA* was higher (*p* < 0.05, Kruskal Wallis test) in MT; moreover, a significant association between *csgA* and PUD (MT and RB) was detected (*p* < 0.05, Kruskal Wallis test) (S2 File).

On the other hand, an "aggregate VF score” (on a scale of 0 to 5) was calculated for each isolate (S4 Table); which, based on the total number of VFG detected, predicts experimental virulence *in vivo* [47]. Overall, the VF aggregate score varied from 0 to 5 among the analyzed isolates. A total of seven isolates proved negative for all the VFG screened, five of which belonged to the group H and two to RB; also, 73, 63 and 36% of isolates from H, RB and MT, respectively, exhibited VF score values ≤ 2. While, 63, 37 and 27% of *E*. *coli* from MT, RB and H, respectively, showed score values ≥ 3 (Table 2); also, only one isolate had the maximum score and belonged to MT group.

**Table 2:** Prevalence of VF score values and phylogenetic classification of *E*. *coli* isolated from H, MT and RB.

	VF		Prevalence of phylogroups (%)*			
Group	Prevalence (%)* Score values		A	B2	B1	D
H (n = 48)	72.9	≤ 2	56.25 (27)^#^	0	8.33 (4)	8.33 (4)
	27.1	≥ 3	27.08 (13)	0	0	0
MT (n = 11)	36.36	≤ 2	27.27 (3)	0	0.09 (1)	0
	63.6	≥ 3	45.45 (5)	0	0.09 (1)	0.09 (1)
RB (n = 38)	63.16	≤ 2	39.47 (15)	0	18.42 (7)	5.26 (2)
	36.84	≥ 3	23.68 (9)	0	10.53 (4)	2.63 (1)

* Among isolates from each animal group;

^#^ number of isolates.

**Fig 4:**
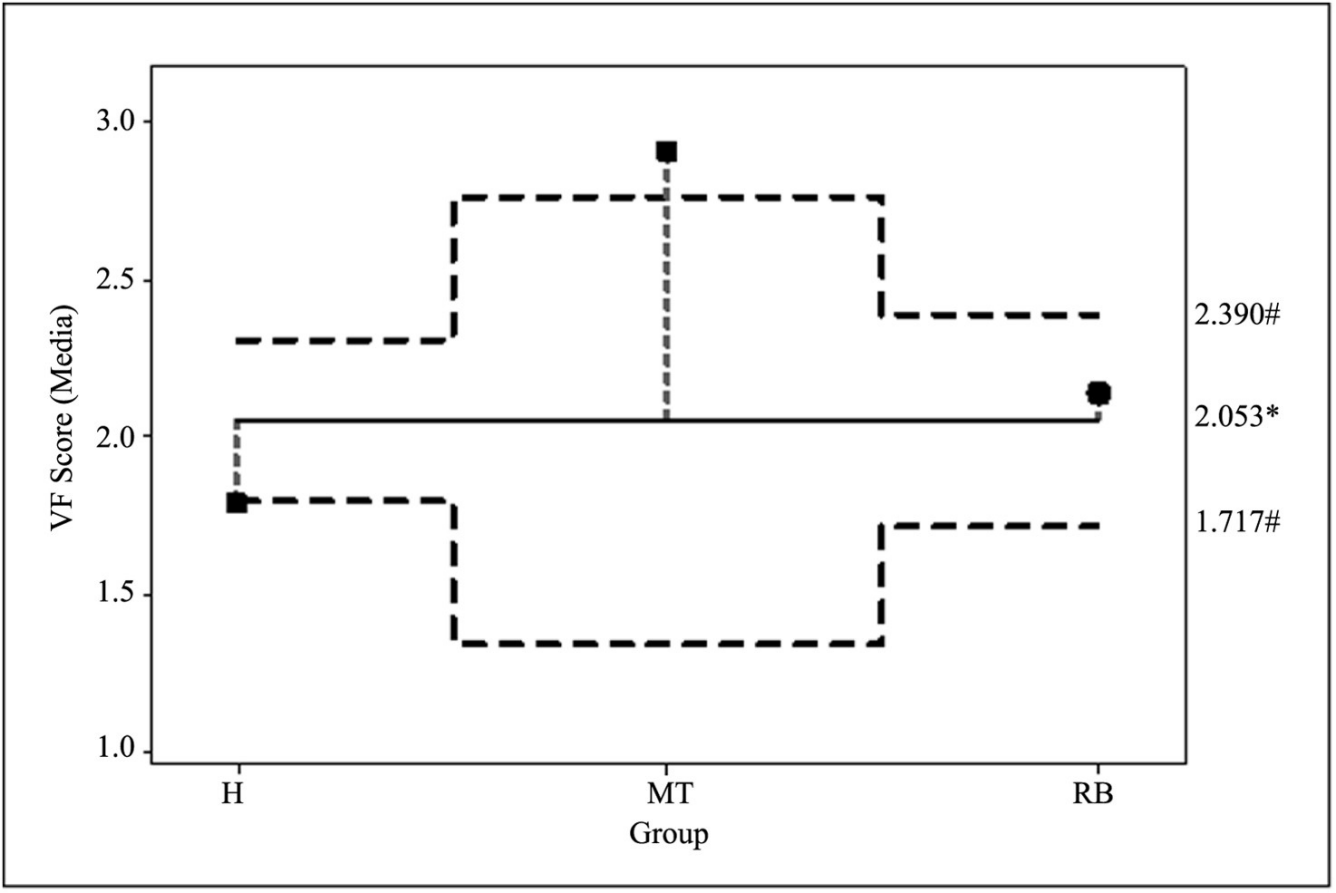
VF score of vaginal *E*. *coli* from (H) healthy heifers, (MT) cows with metritis or endometritis and (RB) repeat breeders. * General media, ^#^ Decision limits. ▀: indicate significant differences (*p* < 0.0001, ANOM).

Moreover, it was possible to determine the association between the VF score and the reproductive profile of the animals; since the analysis of means (ANOM) of the score values obtained for each animal group showed a significant higher score for MT and a lower one for H; however, the mean value calculated for RB was close to the general media (Fig 4).

An analysis of the combinations of phylogenetic groups and VF score yielded 52 patterns, 20 of them were shared by two or more isolates; however, no significant relationship between phylogroup and VF score was observed (Table 2, S4 Table).

**Fig 5:**
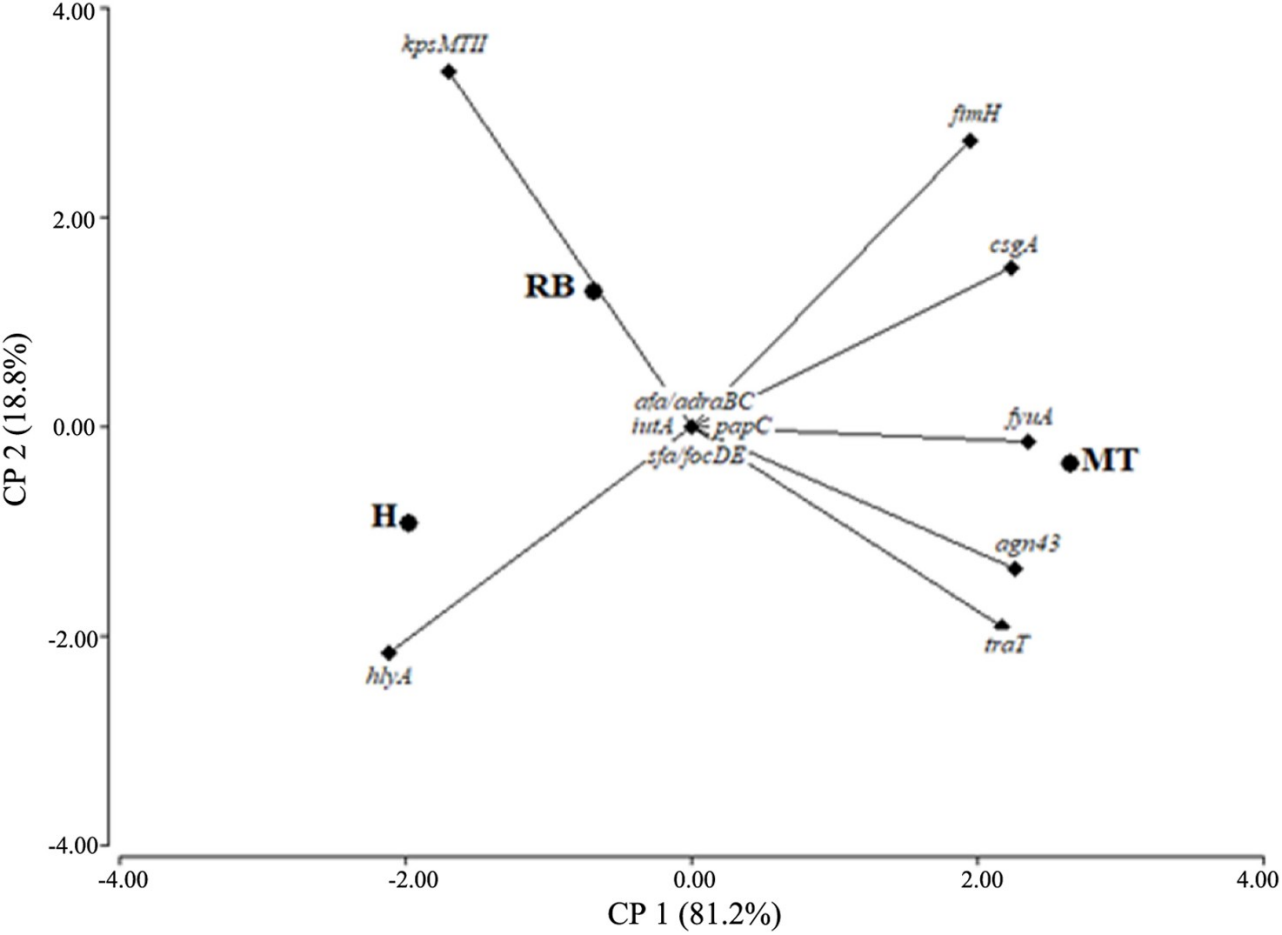
Principal component analysis based on the VFG (virulence factor genes) detection in vaginal *E*. *coli* isolated from healthy heifers (H), cows with metritis or endometritis (MT) and repeat breeders (RB).

A principal component (PC) analysis, based on the presence of extraintestinal VFG, clustered vaginal *E*. *coli* into two groups that incorporate 100% of the cumulative variation: PC1 explained 81.2% of the variation, whereas PC2 explained 18.8%. Plotting PC1 against PC2 for visual evaluation revealed trends in differentiation and clustering of groups with a similar VFG pattern. Thus, several genes, such as *fyuA*, *csgA*, *agn43* and *hlyA* loaded heavily on PC1; the first three were associated to MT and the last one to H, while *kpsMTII* mainly determined the loading for PC2, strongly related to the RB group **(**Fig 5).

### Vaginal *E*. *coli* do not produce hemolysis

None of the *E*. *coli* isolates studied was able to produce α or β-hemolysis.

### *E*. *coli* associated with MT were most mobile

The motility of each isolate was evaluated as the mean of the diameter obtained in three independent experiments. Based on the distribution of the diameters, a scale was defined to categorize the data: immobile (0–5 mm), low motility (5.1–15 mm), medium motility (15.1–30 mm) and high motility (> 30 mm). Furthermore, for the statistical analysis, the isolates were classified as positive (high and medium motility) and negative (low motility or immobile). The results indicated that the prevalence of mobile isolates differed between the animal groups and was significantly higher in the *E*. *coli* populations from MT compared to those from H and RB (91, 52 and 34%, respectively, *p* < 0.005) (Table 3, S3 File).

**Table 3:** Expression of virulence properties in *Escherichia coli* isolated from: Healthy heifers (H), cows with metritis or endometritis (MT) and repeat breeders (RB).

Phenotype (n = 97 *E*. *coli*)	Groups		
	H, n (%)	MT, n (%)	RB, n (%)
Motility	25 (52)^a^	10 (91)^b^	13 (34)^a^
Curli fimbriae/cellulose expression	29 (60)^a^	7 (64)^a^	30 (79)^a^
Biofilm formation	29 (60)^a^	10 (91)^b^	32 (84)^b^
Type 1 fimbriae	19 (40)	5 (45)	12 (32)
Fimbriae P	1 (2)	-	-

^ab^: indicate significant differences between animal groups (*p* < 0.05, T test, 95% CI for the difference).

### Vaginal *E*. *coli* expressed Type 1 fimbriae but not fimbriae P

The prevalence of type 1 fimbriae expression was 45, 40 and 32% in MT, H and RB groups, respectively; instead, the expression of fimbriae P was very low (H) or negative (MT and RB). In neither of the two cases, significant differences according to the reproductive condition were observed (Table 3).

### Vaginal *E*. *coli* produced curli fimbriae and/or cellulose

The prevalence of fimbriae curli and/or cellulose production was of 79, 64 and 60% in RB, MT and H groups, respectively; however, there were no significant differences (Table 3).

### Biofilm formation ability was higher for *E*. *coli* associated with MT

**Fig 6:**
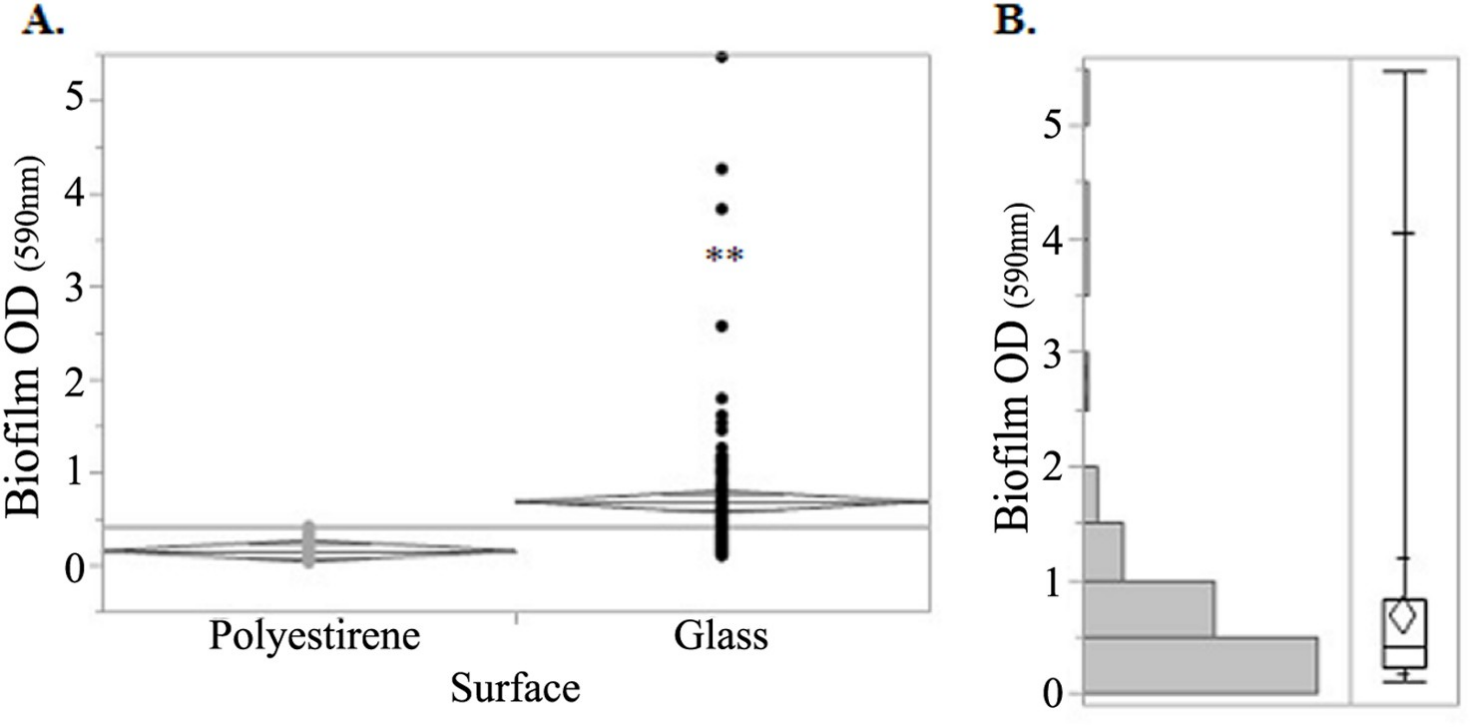
**A**. Biofilm formation of vaginal *E*. *coli* isolates on polystyrene and glass. **B**. Distribution of biofilm production values on glass: median value = 0.42; quartiles: Q1 = 0.24 and Q3 = 0.82, which define the IQ (interquartile interval) of the values of average capacity of biofilm formation. **: indicate significant differences (*p* < 0.0001, Student's t-test).

The biofilm formation values were significantly (*p* ≤ 0.0001, Student's t-test) affected by the surfaces where it developed (glass or polystyrene) (Fig 6A). Based on the greater amplitude of the obtained values on glass, it was decided to continue evaluating the biofilm production capacity on this surface. The results were categorized considering the interquartile ranges (OD_590 nm_) obtained for all the tested isolates: low (< 0.24), medium (0.24 to 0.82) and high (> 0.82) (Fig 6B). The expression of this property was significantly higher in the *E*. *coli* population from MT and RB than H (*p* < 0.03, T test, 95% CI for the difference) (Table 3, S3 File).

### Biofilm and curli fimbriae/cellulose expression were associated to *E*. *coli* from MT cows

**Fig 7:**
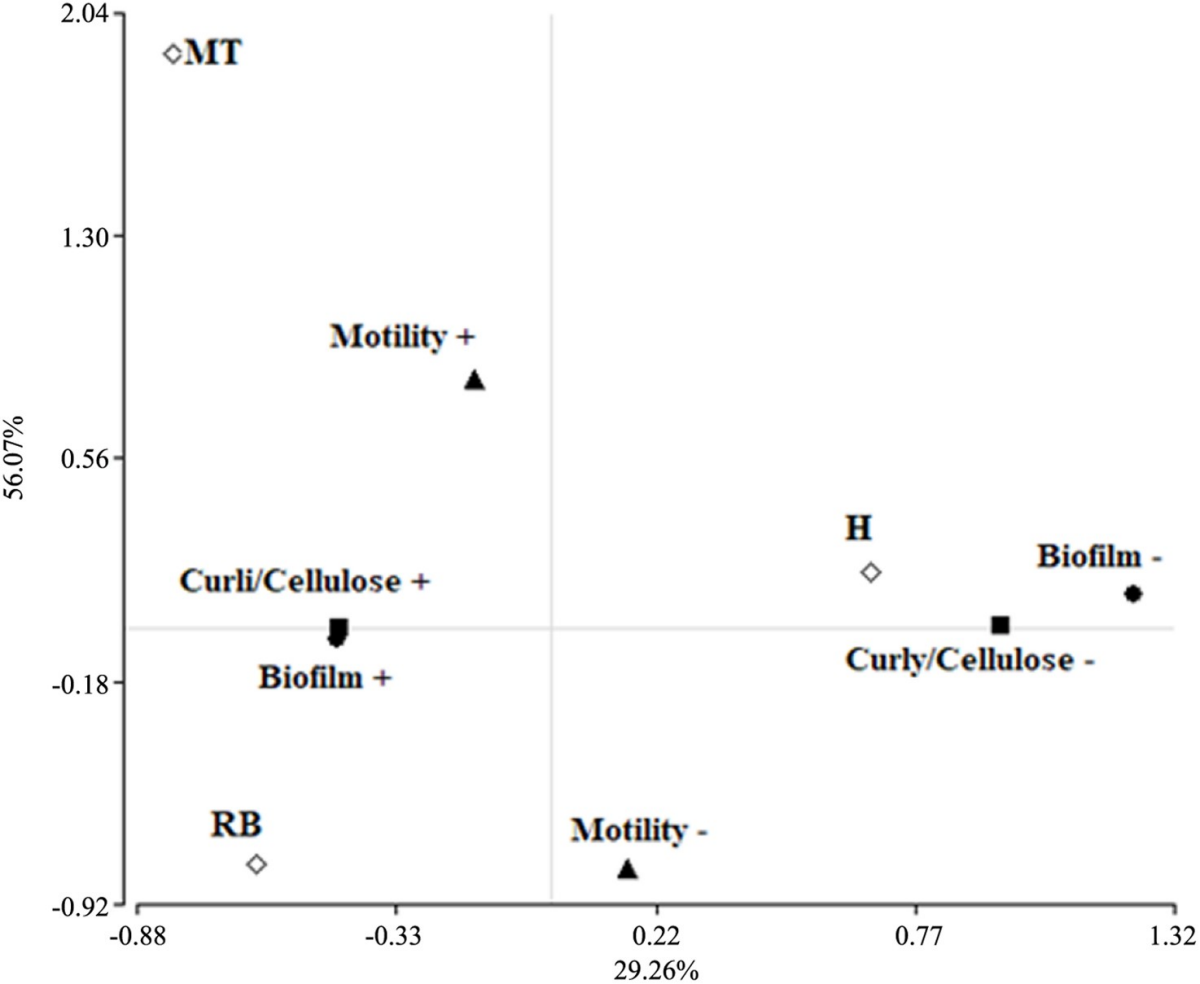
Analysis of correspondence: Biofilm formation, expression of curli fimbriae/cellulose and motility of vaginal *E*. *coli* isolated from (H) healthy heifers, (MT) cows with metritis or endometritis and (RB) repeat breeders. The contribution to Chi-square is indicated in brackets.

The correspondence analysis performed between biofilm formation, curli fimbriae/cellulose production, motility and the reproductive profiles of the sampled animals, showed that the *E*. *coli* isolated from H were associated to negative expression of biofilm, fimbriae curli/cellulose and motility. On the other hand, the positive values for these properties were associated with MT, while the RB group showed positive association with biofilm formation and curli fimbriae/cellulose production but not with motility (Fig 7).

## Discussion

The present study describes, for the first time, the potential pathogenicity of vaginal *E*. *coli* populations from healthy heifers (H) and repeat breeders (RB) compared with those from cows with postpartum uterine disease (MT).

Even though *E*. *coli* has been described as normal microbiota member of the bovine reproductive tract (BRT) in healthy heifers [20], it has a decisive role in the establishment of metritis [12, 13, 27]. The presence of *E*. *coli* in the early postpartum uterus is associated with inflammation, purulent vulvar discharge, metritis and problems in the restoration of estrus [15]; however, there is still no consensus on the virulence profile of *E*. *coli* strains with endometrial pathogenic features [2, 4, 14, 26].

Several reports have approached the isolation of bacteria colonizing the uterus in postpartum of metritic and healthy cows [25]; nerveless, to date, no scientific work had isolated and characterized native vaginal *E*. *coli* from RB cows, as performed in the present study.

Genome plasticity is the main driver of *E*. *coli* phenotypic diversity and evolution [48]; thus, this dynamism is responsible for the relevant differences observed among the pathogenic strains associated with diseases [49]. In the present study, the genomic diversity among vaginal *E*. *coli* was assessed through the ERIC-PCR technique as previously applied for animal samples taken from different mucosae [50, 51].

The findings of this manuscript show strong association between genetic diversity and postpartum uterine disease. An interesting parallel to the loss of genetic diversity in the population of *E*. *coli*, is the finding of a lower microbial diversity in early postpartum vagina of dairy cows that at 21 DPP developed postpartum endometritis [52]. Similar results were observed in uterus [23]. One may speculate that the loss of genetic diversity is likely a commonplace phenomenon in bacterial populations of cows suffering PUD. A selection pressure exerted by exposure to antimicrobial and sustained inflammatory response in PUD cows [53] may drive the loss of genetic diversity while selecting variants capable of surviving such challenges.

Phylogenetic analysis has been used to evaluate the evolutionary origins of pathogenic *E*. *coli* isolated from postpartum bovine uterus, where the predominant groups were B1, A and D [2, 14]. However, our results showed that the phylogenetic conformation of native *E*. *coli* populations of bovine vagina was mainly constituted by the phylogroup A, B1 and, to a lesser extent, by D; nevertheless, no isolate was classified as B2. This discrepancy could be explained by the fact that we evaluated *E*. *coli* populations not only from postpartum cows, but also from healthy heifers; which were associated with phylogroup A. It seems that niche-partitioning of *E*. *coli* occurs between healthy heifers and cows with reproductive disorders as a result of selective pressure, especially in RB cows since phylogenetic group B1 was significantly more represented in this last group. Likewise, it is important to keep in mind that our study focused on vaginal isolates and not uterine like the other researches [2, 14]; this fact may also help to clarify the differences among results. Additional studies should be performed to extend the investigation of the characteristics of the B1 populations that reside in the vagina in order to determine if they have a role in the emergence of endometrial pathogenic *E*. *coli* strains. A recent report showed that vaginal and uterine microbial communities are similar during the early postpartum period [52]. While these communities differentiated early in the healthy cow, a delayed differentiation was observed in cows that developed postpartum endometritis. So, at least in the PUD cows, it is theoretically possible that vaginal populations contribute to uterine populations of *E*. *coli*.

Several approaches have been performed in order to describe pathogenic *E*. *coli* in the BRT; Silva et al. [14] evaluated 15 VFG in uterine *E*. *coli*, nerveless no association was found between them and metritis. In contrast, Sheldon et al. [2] studied 17 VFG and concluded that *E*. *coli* associated with postpartum uterine infections possess the *fyuA* gene; while Bicalho et al. [4] evaluated 32 VFG and reported that 6 of them (*fimH*, *hlyA*, *cdt*, *kpsMT II*, *ibeA* and *astA*) were associated with postpartum uterine disease. On the other hand, Kassé et al. [26] studied 40 VFG and observed that only *hra1* and *kpsMT II* were associated with uterine infections. It is probable that the dissidence in their conclusions comes from the fact that these studies did not evaluate the same VFG. In addition, even though Kassé et al. [26] included a greater number of genes in their study, they used a different methodology than the other groups. Another possibility could be that the distribution of VF varies between farms and regions [2, 4, 14, 26]. In the present study, we evaluated samples taken from three dairy farms from Tucumán province (North West of Argentina), therefore our results may differ with the cited scientific works since they took place in North America and Europe; moreover, we studied populations resident in vagina and not in uterus, in order to define native *E*. *coli* with risk profile for postpartum infection.

None of the 97 vaginal isolates studied proved positive for the typical diarrheagenic *E*. *coli* genes in agreement with the findings obtained by Silva et al. [14]. These results were expected since both, commensal *E*. *coli* and ExPEC strains have a low or negative prevalence of intestinal VFG [54]. However, 7 of the 11 tested extraintestinal VFG were detected and the most prevalent was *fimH* in agreement with Bicalho et al. [4] and Kassé et al [26] findings. Also, Bicalho et al. [4] indicated that this gene is an important predictor of bovine metritis and endometritis. In our study, the expression of fimbriae type 1 showed a higher prevalence among isolates from cows with metritis or endometritis (MT). This result is particularly interesting since the adhesion of pathogenic *E*. *coli* to endometrial cells depends on the expression of this gene [2]. In addition, only one isolate from healthy heifers (H) expressed mannose resistant hemagglutination. However, it is not possible to affirm that this last result is due to fimbriae P expression, since *papC*, which code for the outer membrane usher protein, was not detected in any of the vaginal *E*. *coli*. Nevertheless, these results may indicate the expression of other mannose resistant fimbriae; whose genes were not evaluated in the present study, such as fimbriae G, M-agglutinin or non-fimbrial adhesin 1 [36]. Future studies are necessary to elucidate which fimbriae could be associated with the colonization of the vaginal mucosal surface in cows. Biofilm formation contributes to the pathogenicity of several microorganisms and is considered an important step in the pathogenesis of ExPEC [55, 56]. In the present study, motility and biofilm formation were significantly higher in *E*. *coli* isolated from MT cows; moreover, this last property was also significantly higher in the bacterial population from RB compared to those from the H group. Curli production and *agn43* expression play a key role in the initial stages of the *E*. *coli* biofilm formation [57]; therefore, in this work, we evaluated the presence of *agn43* and *csgA* (the major subunit of the fimbriae curli) and demonstrated a high prevalence of both genes; being *csgA* significantly associated with RB and MT cows. This agrees with the phenotypic characterization that showed a positive association between biofilm formation and positive curli/cellulose fimbriae production with PUD (MT and RB). These findings are relevant because the biofilm extracellular matrix of *E*. *coli* is mainly composed of curli fimbriae and cellulose [58]. On the contrary, these properties do not seem to be predominant among the *E*. *coli* populations from healthy heifers.

Uterine infection presupposes bacterial survival and microcolonies formation during colonization progression, which requires bacterial resistance against the host's defense mechanisms [23]. In this context, we investigated the presence of the *traT* gene, which codes for the R6-5 plasmid-specified outer membrane protein that mediates resistance to bacterial killing by serum. Even though there was a high prevalence of *traT*, no differences regarding the detection of this gene were observed between *E*. *coli* populations from H, MT and RB.

The higher prevalence of *fyuA* detected in MT agrees with the observations previously described by Sheldon et al. [2], who showed that this VFG was found in uterine *E*. *coli* from cows with uterine diseases but not from healthy ones. Considering that *fyuA* encodes the outer membrane protein yersiniabactin, that is important for the iron uptake as wells as for biofilm formation in UPEC [59], complementary studies with a greater number of cows should be carried out to confirm the relevance of this VFG in the pathogenesis of postpartum uterine diseases.

In the phenotypic characterization no isolate capable of producing hemolysis was observed; however, *hlyA* was found in *E*. *coli* from RB and H, but not in MT in discrepancy with Bicalho et al. [4], who observed a significant association between *hlyA* and the occurrence of metritis. Therefore, we cannot deduce a role for *hlyA* in the establishment of infection.

Capsular structures promote bacterial survival during infection; thus, *kpsMT II* has been associated with *E*. *coli* responsible for cellulitis, in chickens [60], as well as with UTI, in women [61, 62]. Both Kassé et al. [26] and Bicalho et al. [4] reported an association between this virulence gene and the incidence of postpartum bovine metritis. However, in our study, the principal components analysis exposed an association between *kpsMT II* and RB cows, which should be a potential link with the etiology of the RB syndrome.

Although we found no differences in the VFG score among phylogroups, relevant results were observed when comparing this score and the reproductive profile of the animals. Isolates from MT showed a significantly higher score whereas those from the H group had a significantly lower one. Once more our observations suggest the existence of a selection of *E*. *coli* strains with several VFG which may increase the risk of uterine disease and reproductive failure.

To our knowledge, these results for the first time propose that exploring the virulence repertory which includes VFG or other bacterial characteristics in vaginal *E*. *coli* associated with phylogenetic B1, might be helpful to understand the pathogenesis behind postpartum uterine diseases. Based on the analyzes and associations found in the present study, future research focusing on *E*. *coli* from phylogroup B1 that carriers the genes *fyuA* and *csgA*, mobile, with an elevated curli fimbriae/cellulose expression and biofilm formation capacity, should be performed to define risk patterns associated with uterine disease (MT and RB).

Although to date there is no etiological agent associated with the RB syndrome; taking into consideration that *E*. *coli* is capable of altering the endocrine function of the BRT by disturbing ovulation, as well as the embryo viability and that cows with subclinical endometritis have high serum concentrations of LPS 40 to 60 days after parturition [13, 18, 19], it is likely that this microorganism may participate in the pathogenesis of the RB syndrome. Complementary studies should be done to evaluate the implication of *E*. *coli* in this syndrome. Our findings showed for the first time, a significant association among RB cows with *E*. *coli* from phylogenetic group B1, with the presence of *fyuA*, *csgA*, and *kpsMT II*, as well as a higher potential for curli fimbriae/cellulose expression and biofilm formation. These virulence attributes could be used for early identification of cows with an elevated risk level for developing this syndrome.

## Conclusion

From these results, it can be concluded that vaginal *E*. *coli* from animals with metritis or endometritis or repeat breeders possess VFG that could confer ability to colonize the bovine genitalia. We have identified for the first time, the phylogenic arrangement for native *E*. *coli* populations in bovine vagina, in phylogroups A and B1, where this last group is associated with uterine disease postpartum.

Trying to identify virulence feature that could be used as markers for detecting endometrial pathogenic *E*. *coli* populations in vagina, we observed that strains from phylogroup B1, that possess VFG *fyuA* and *csgA* as well as elevated motility, curli fimbriae/cellulose expression and biofilm formation capacity, may play a prevalent role in the pathogenesis of postpartum disorders. Specifically, regarding the RB syndrome, vaginal populations of B1 *E*. *coli* that carry *fyuA*, *csgA* and *kpsMT II*, curli fimbriae/cellulose biosynthesis and with higher biofilm formation attributes may have a pathogenic potential for causing this syndrome.

Future research focusing on the detection of these strains in the vaginal microbiota of cows with postpartum uterine diseases should be done. The control of their presence in vagina would reduce the risk that they access the uterus during the postpartum period.

## Supporting information

S1 Fig Phylogroups prevalence shown by bovine vaginal *E*. *coli*. Multiplex amplification of DNA from various *E*. *coli* strains isolated from bovine vagina using *chuA*, *yjaA* and TspE4.C2 primers. Lane 13: 50-bp DNA ladder (Thermo Fisher Scientific, Waltham, Massachusetts, USA).
(TIF)

S2 Fig **a. Virulence factor gene (VFG) detection in vaginal *E*. *coli* strains from Holstein heifers and cows**. The presence of the following VFGs was assessed by PCR: *fimH* and *hlyA*. **b. Virulence factor gene (VFG) detection in vaginal *E*. *coli* strains from Holstein heifers and cows**. The presence of the following VFGs was assessed by PCR *kpsMTII*. **c. Virulence factor gene (VFG) detection in vaginal *E*. *coli* strains from Holstein heifers and cows**. The presence of the following VFGs was assessed by PCR: *papC* and *fyuA*. **d. Virulence factor gene (VFG) detection in vaginal *E*. *coli* strains from Holstein heifers and cows**. The presence of the following VFGs was assessed by PCR *traT*. **e. Virulence factor gene (VFG) detection in vaginal *E*. *coli* strains from Holstein heifers and cows**. The presence of the following VFGs was assessed by PCR: *csgA* and *agn43*.
(TIF)

S3 Fig (JPG)

S4 Fig (PSD)

S5 Fig (PSD)

S1 Table Richness of fingerprint (ERIC-PCR) profiles of vaginal *E*. *coli* in animals from H and PUD groups. (DOCX)

S2 Table Raw data of phylogenetic classification of vaginal *E*. *coli* isolated from H, MT and RB. (XLSX)

S3 Table Raw data of VFG PCR products of vaginal *E*. *coli* isolated from H, MT and RB. (DOCX)

S4 Table Virulence and phylogenetic profile of vaginal *E*. *coli* isolated from H, MT and RB. (DOCX)

S1 File **a. Raw dendograms generated by Bionumerics (Applied Maths, Sint-Martens-Latem, Belgium) software of ERIC-PCR (ERIC = enterobacterial repetitive intergenic consensus) fingerprints of 48 bovine vaginal *E*. *coli* from healthy heifers**. The original gel images were uploaded to the Bionumeric software where data from ERIC-PCR were combined into a composite data set; the dendrogram obtained was used to analyze the degree of similarity between *E*. *coli* isolates. **b. Raw dendograms generated by Bionumerics (Applied Maths, Sint-Martens-Latem, Belgium) software of ERIC-PCR (ERIC = enterobacterial repetitive intergenic consensus) fingerprints of 49 bovine vaginal *E*. *coli* from cows with uterine postpartum diseases (UPD). The original gel images were uploaded to the Bionumeric software where data from ERIC-PCR were combined into a composite data set; the dendrogram obtained was used to analyze the degree of similarity between *E*. *coli* isolates**.
(PDF)

S2 File Statistical analysis of virulence factor gene (VFG) detection in vaginal *E*. *coli* strains among animal sampling groups. (PDF)

S3 File Statistical analysis of the expression of virulence properties in vaginal *E*. *coli* strains among animal sampling groups. (DOCX)
