# Supplementary figures and images for: Characterization of native Escherichia coli populations from bovine vagina of healthy heifers and cows with postpartum uterine disease
Source: PLoS One. 2020 Jun 1;15(6):e0228294. doi: 10.1371/journal.pone.0228294 (PMC7263596; doi:10.1371/journal.pone.0228294)

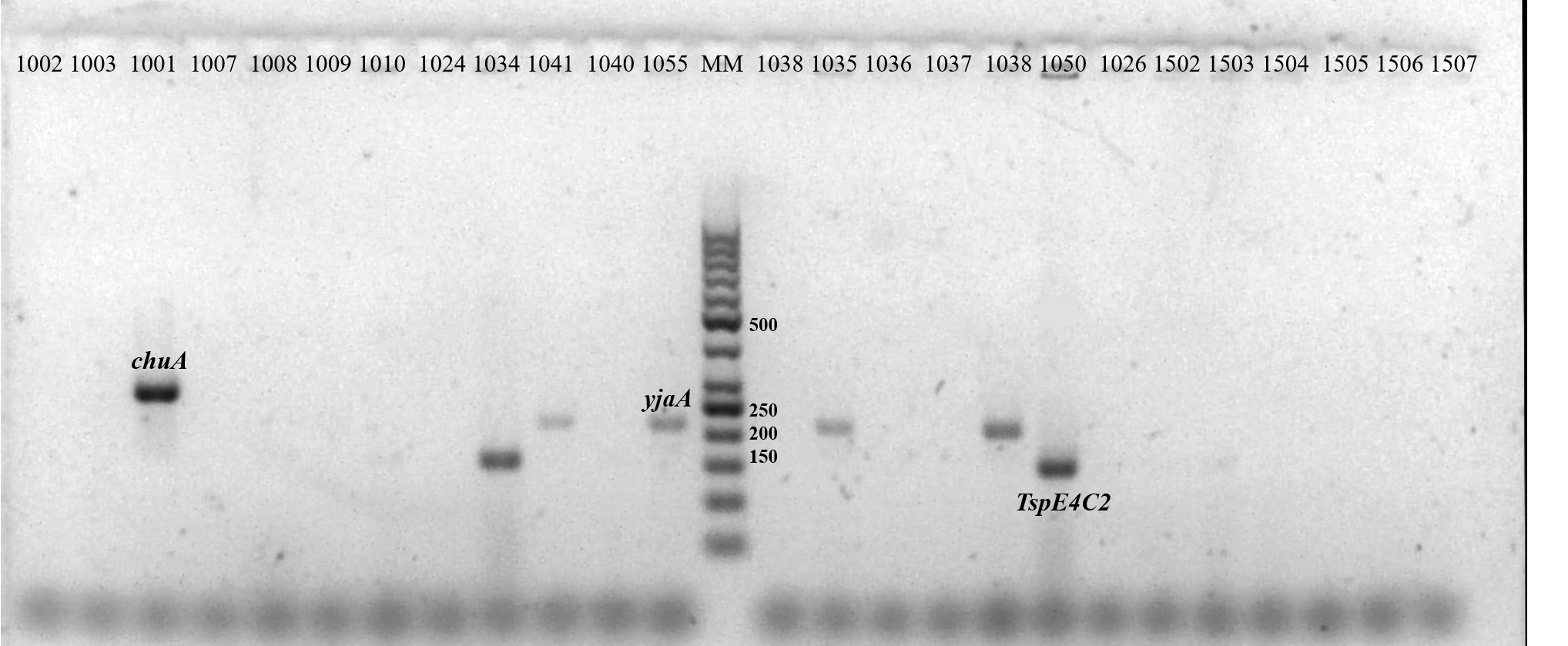

Supplement: S1 Fig — Multiplex amplification of DNA from various E. coli strains isolated from bovine vagina using chuA, yjaA and TspE4.C2 primers. Lane 13: 50-bp DNA ladder (Thermo Fisher Scientific, Waltham, Massachusetts, USA). (TIF) [file pone.0228294.s001.tif]

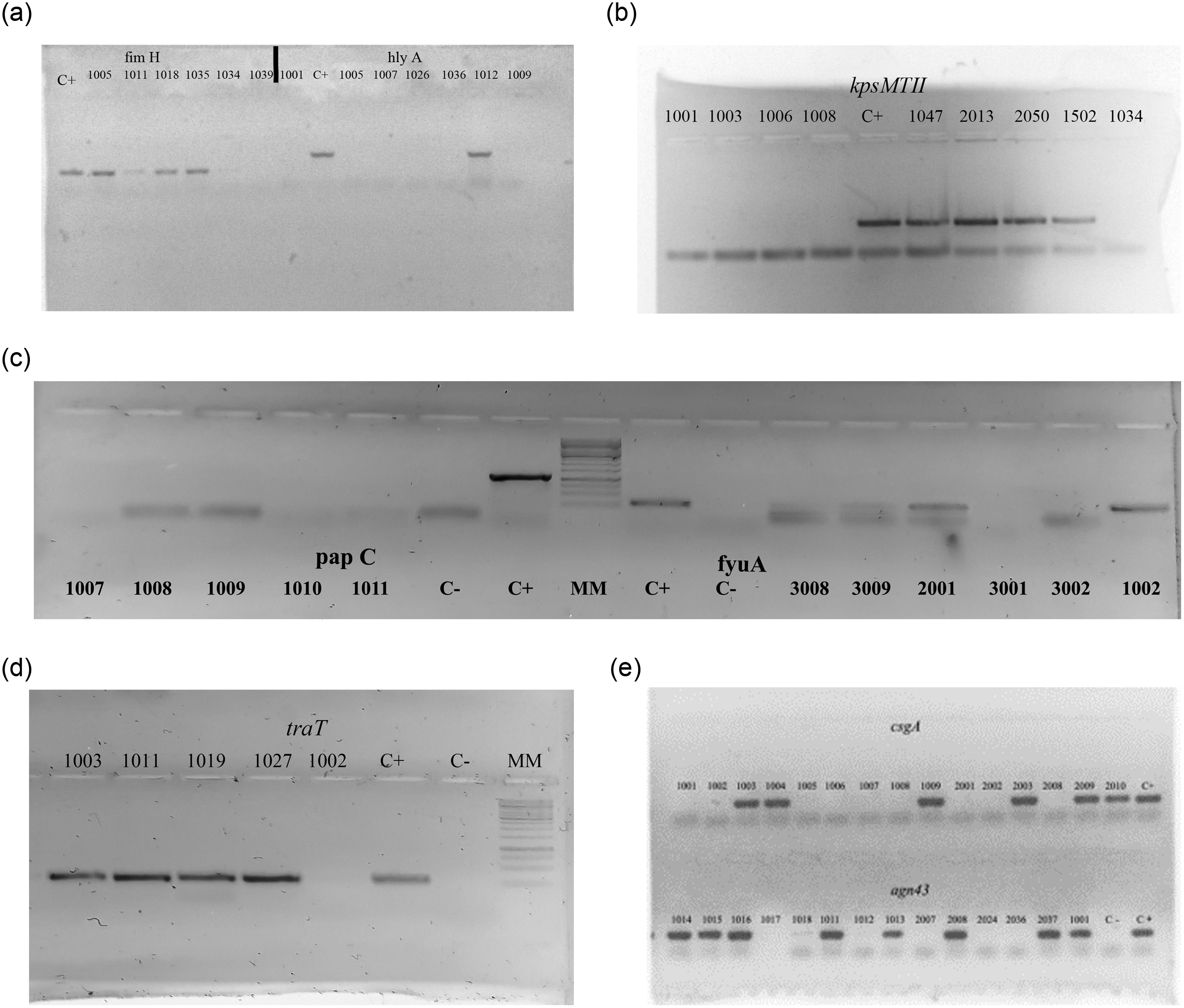

Supplement: S2 Fig — a. Virulence factor gene (VFG) detection in vaginal E. coli strains from Holstein heifers and cows. The presence of the following VFGs was assessed by PCR: fimH and hlyA. b. Virulence factor gene (VFG) detection in vaginal E. coli strains from Holstein heifers and cows. The presence of the following VFGs was assessed by PCR kpsMTII. c. Virulence factor gene (VFG) detection in vaginal E. coli strains from Holstein heifers and cows. The presence of the following VFGs was assessed by PCR: papC and fyuA. d. Virulence factor gene (VFG) detection in vaginal E. coli strains from Holstein heifers and cows. The presence of the following VFGs was assessed by PCR traT. e. Virulence factor gene (VFG) detection in vaginal E. coli strains from Holstein heifers and cows. The presence of the following VFGs was assessed by PCR: csgA and agn43. (TIF) [file pone.0228294.s002.tif]

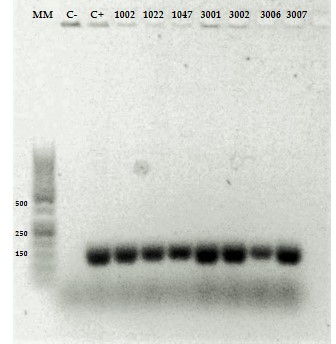

Supplement: S3 Fig — (JPG) [file pone.0228294.s003.jpg]

(a)

eric vq tambo

eric vq tambo

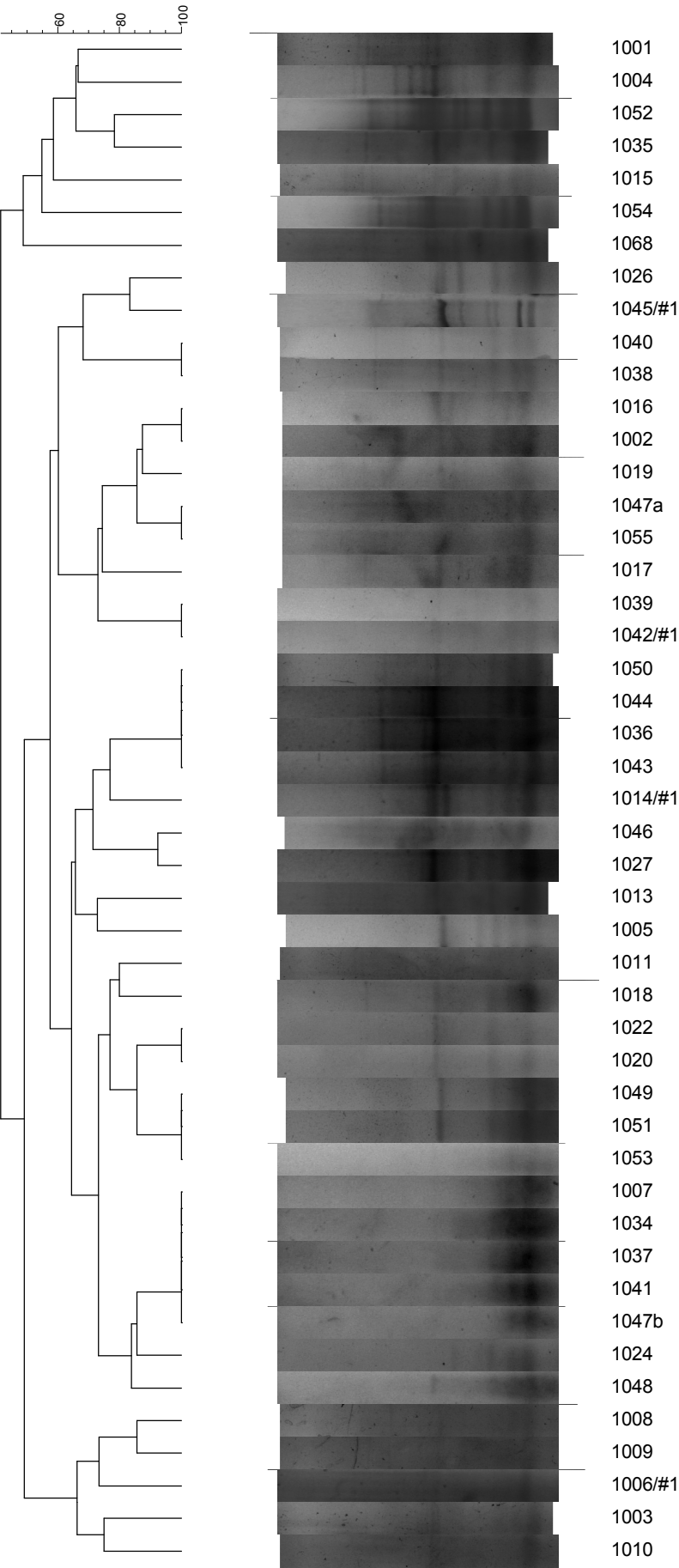

(b)

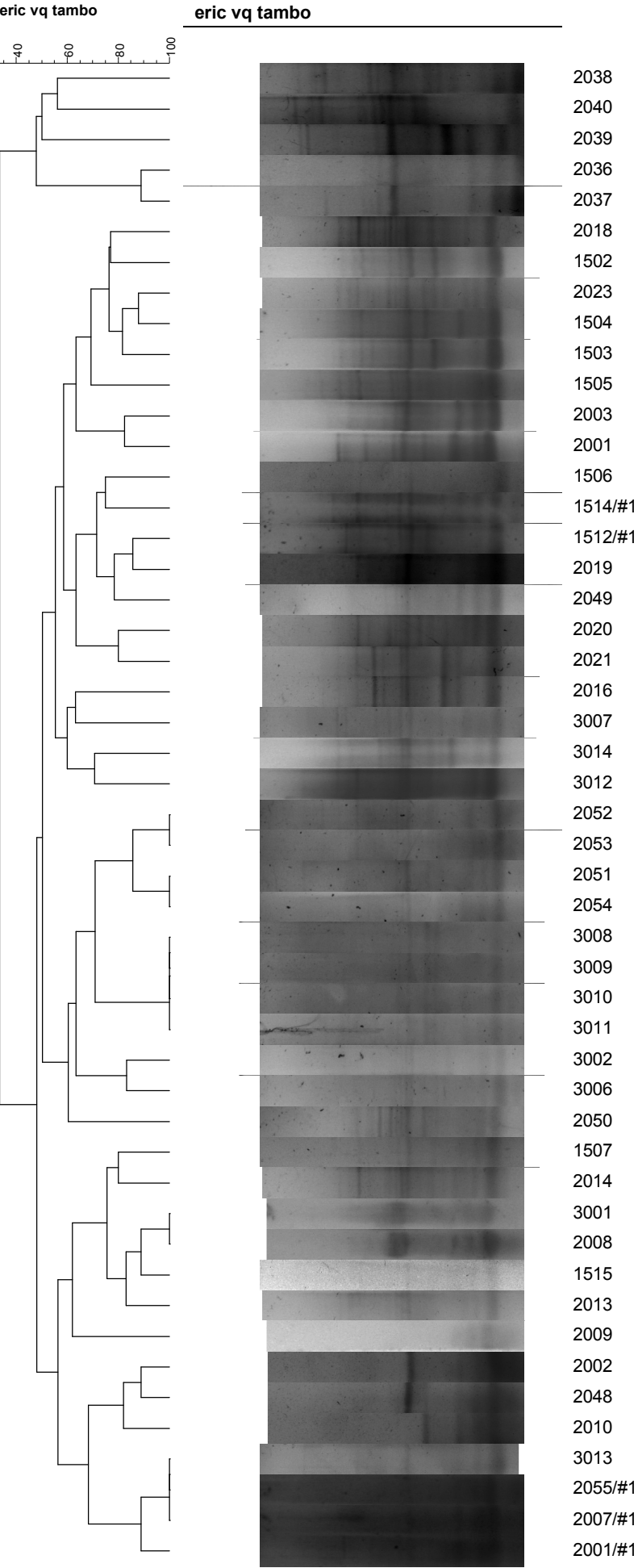

Supplement: S1 File — a. Raw dendograms generated by Bionumerics (Applied Maths, Sint-Martens-Latem, Belgium) software of ERIC-PCR (ERIC = enterobacterial repetitive intergenic consensus) fingerprints of 48 bovine vaginal E. coli from healthy heifers. The original gel images were uploaded to the Bionumeric software where data from ERIC-PCR were combined into a composite data set; the dendrogram obtained was used to analyze the degree of similarity between E. coli isolates. b. Raw dendograms generated by Bionumerics (Applied Maths, Sint-Martens-Latem, Belgium) software of ERIC-PCR (ERIC = enterobacterial repetitive intergenic consensus) fingerprints of 49 bovine vaginal E. coli from cows with uterine postpartum diseases (UPD). The original gel images were uploaded to the Bionumeric software where data from ERIC-PCR were combined into a composite data set; the dendrogram obtained was used to analyze the degree of similarity between E. coli isolates. (PDF) [file pone.0228294.s010.pdf]
