# Supplementary material for: Characterization of native Escherichia coli populations from bovine vagina of healthy heifers and cows with postpartum uterine disease
Source: PLoS One. 2020 Jun 1;15(6):e0228294. doi: 10.1371/journal.pone.0228294 (PMC7263596; doi:10.1371/journal.pone.0228294)
Supplement: S1 Table — (DOCX) [file pone.0228294.s006.docx]

| **S1 Table.** Richness of fingerprint (ERIC-PCR) profiles of vaginal *E. coli* in animals from H and PUD groups. | | | | | | | | | |
| --- | --- | --- | --- | --- | --- | --- | --- | --- | --- |
| ***Group** |  | **sampled animals** | | | **animals with ^#^***E. coli* **+** | **^‡^Different profiles per animal** | | **Number of animal** | |
| H |  | 48 | 22.9 % (n = 11) | | | 1 | 2 | |  |
|  |  |  |  | | | 2 | 1 | |  |
|  |  |  | |  | | 3 | 1 | |  |
|  |  |  | | | | 4 | 0 | |  |
|  |  |  | | | | 5 | 1 | |  |
|  |  |  | | | | 6 | 4 | |  |
|  |  |  | | | | 7 | 2 | |  |
| PUD |  | 49 | 42.85 % (n = 21) | | | 1 | 9 | |  |
|  |  |  |  | | | 2 | 4 | |  |
|  |  |  |  | | | 3 | 5 | |  |
|  |  |  |  | | | 4 | 2 | |  |
|  |  |  |  | | | 5 | 1 | |  |
|  |  |  |  | | | 6 | 0 | |  |
|  |  |  |  | | | 7 | 0 | |  |

**^*^** Animal groups were healthy heifers (H) and cows with postpartum uterine diseases (PUD); **^#^**animals with positive cultures for *E. coli*; **^‡^** fingerprint profiles with similarity (Pearson coefficient) ˂ 90 %.
