# Supplementary material for: Characterization of native Escherichia coli populations from bovine vagina of healthy heifers and cows with postpartum uterine disease
Source: PLoS One. 2020 Jun 1;15(6):e0228294. doi: 10.1371/journal.pone.0228294 (PMC7263596; doi:10.1371/journal.pone.0228294)
Supplement: S4 Table — (DOCX) [file pone.0228294.s009.docx]

| **S4 Table. Virulence and phylogenetic profile of vaginal *E. coli* isolated from H, MT and RB.** | | | | |
| --- | --- | --- | --- | --- |
| **VFG score** | **Group** | **Phylogroup** | **VFG** | **Nº** |
| 0 | H**^*^** | A | - | 3 |
|  | H | B1 | - | 2 |
|  | RB | A | - | 1 |
|  | RB | B1 | - | 1 |
| 1 | H | A | *fimH* | 11 |
|  | H | A | *agn43* | 1 |
|  | H | B1 | *fimH* | 2 |
|  | H | D | *fimH* | 1 |
|  | RB**^#^** | A | *fimH* | 5 |
|  | RB | B1 | *fimH* | 1 |
|  | RB | B1 | *csgA* | 1 |
| 2 | H | A | *csgA, agn43* | 1 |
|  | H | A | *agn43, traT* | 1 |
|  | H | A | *fimH, csgA* | 4 |
|  | H | A | *fimH, agn43* | 3 |
|  | H | A | *fimH, kpsMTII* | 1 |
|  | H | A | *fimH, traT* | 2 |
|  | H | D | *fimH, agn43* | 2 |
|  | H | D | *fimH, traT* | 1 |
|  | RB | A | *fimH, csgA* | 6 |
|  | RB | A | *fimH, agn43* | 3 |
|  | RB | B1 | *fimH, csgA* | 2 |
|  | RB | B1 | *fimH, agn43* | 1 |
|  | RB | B1 | *fimH, fyuA* | 1 |
|  | RB | D | *fimH, agn43* | 1 |
|  | RB | D | *agn43, fyuA* | 1 |
|  | MT**^†^** | A | *fimH, fyuA* | 2 |
|  | MT | A | *csgA, fyuA* | 1 |
|  | MT | D | *fimH, agn43* | 1 |
| 3 | H | A | *fimH, csgA, agn43* | 1 |
|  | H | A | *fimH, csgA, traT* | 2 |
|  | H | A | *fimH, agn43, hlyA* | 4 |
|  | H | A | *fimH, agn43, kpsMTII* | 1 |
|  | H | A | *fimH, agn43, traT* | 2 |
|  | H | A | *fimH, agn43, fyuA* | 1 |
|  | RB | A | *fimH, csgA, agn43* | 1 |
|  | RB | A | *fimH, csgA, kpsMTII* | 2 |
|  | RB | A | *fimH, csgA, traT* | 2 |
|  | RB | A | *fimH, agn43, traT* | 1 |
|  | RB | A | *fimH, agn43, fyuA* | 1 |
|  | RB | B1 | *fimH, csgA, agn43* | 1 |
|  | RB | B1 | *fimH, agn43, traT* | 1 |
|  | RB | B1 | *fimH, hlyA, traT* | 1 |
|  | RB | D | *fimH, csgA, agn43* | 1 |
|  | MT | A | *fimH, csgA, agn43* | 2 |
|  | MT | A | *fimH, csgA, fyuA* | 1 |
|  | MT | A | *fimH, agn43, traT* | 1 |
|  | MT | B1 | *fimH, csgA, traT* | 1 |
| 4 | H | A | *fimH, csgA, agn43, traT* | 2 |
|  | RB | A | *fimH, agn43, traT, fyuA* | 1 |
|  | RB | A | *fimH, csgA, agn43, traT* | 1 |
|  | RB | B1 | *fimH, agn43, kpsMTII, fyuA* | 1 |
|  | MT | A | *fimH, csgA, agn43, traT* | 1 |
| 5 | MT | B1 | *fimH, csgA, agn43, traT, fyuA* | 1 |
| ^*^H: healthy heifers; ^#^RB: repeat breeders; ^†^MT: cows with metritis or endometritis; ‡ number of isolates sharing each profile. | | | | |
