# Supplementary material for: Characterization of native Escherichia coli populations from bovine vagina of healthy heifers and cows with postpartum uterine disease
Source: PLoS One. 2020 Jun 1;15(6):e0228294. doi: 10.1371/journal.pone.0228294 (PMC7263596; doi:10.1371/journal.pone.0228294)
Supplement: S2 File — (PDF) [file pone.0228294.s011.pdf]

Nueva tabla_1 : 11/5/2018 - 7:02:13 PM - [Versión : 3/31/2015]

Prueba de Kruskal Wallis

Variable	Group	N	Medias	D.E.	Medianas	H	p
fim H	Vmt	11	0.91	0.30	1.00	0.31	0.6445
fim H	Vq	48	0.83	0.38	1.00		
fim H	Vr	38	0.89	0.31	1.00		

Variable	Group	N	Medias	D.E.	Medianas	H	p
pap C	Vmt	11	0.00	0.00	0.00	0.00	>0.9999
pap C	Vq	48	0.00	0.00	0.00		
pap C	Vr	38	0.00	0.00	0.00		

Variable	Group	N	Medias	D.E.	Medianas	H	p
sfa/foc DE	Vmt	11	0.00	0.00	0.00	0.00	>0.9999
sfa/foc DE	Vq	48	0.00	0.00	0.00		
sfa/foc DE	Vr	38	0.00	0.00	0.00		

Variable	Group	N	Medias	D.E.	Medianas	H	p
afa/adra BC	Vmt	11	0.00	0.00	0.00	0.00	>0.9999
afa/adra BC	Vq	48	0.00	0.00	0.00		
afa/adra BC	Vr	38	0.00	0.00	0.00		

Variable	Group	N	Medias	D.E.	Medianas	H	p
csgA	Vmt	11	0.64	0.50	1.00	6.61	0.0079
csgA	Vq	48	0.21	0.41	0.00		
csgA	Vr	38	0.45	0.50	0.00		

Variable	Group	N	Medias	D.E.	Medianas	H	p
iutA	Vmt	11	0.00	0.00	0.00	0.00	>0.9999
iutA	Vq	48	0.00	0.00	0.00		
iutA	Vr	38	0.00	0.00	0.00		

Variable	Group	N	Medias	D.E.	Medianas	H	p
Agn 43	Vmt	11	0.55	0.52	1.00	0.65	0.6384
Agn 43	Vq	48	0.40	0.49	0.00		
Agn 43	Vr	38	0.39	0.50	0.00		

Variable	Group	N	Medias	D.E.	Medianas	H	p
hly	Vmt	11	0.00	0.00	0.00	0.30	0.3564
hly	Vq	48	0.08	0.28	0.00		
hly	Vr	38	0.03	0.16	0.00		

Variable	Group	N	Medias	D.E.	Medianas	H	p
Kps MT II	Vmt	11	0.00	0.00	0.00	0.19	0.5315
Kps MT II	Vq	48	0.04	0.20	0.00		
Kps MT II	Vr	38	0.08	0.27	0.00		

Variable	Group	N	Medias	D.E.	Medianas	H	p
traT	Vmt	11	0.36	0.50	0.00	0.83	0.4406
traT	Vq	48	0.21	0.41	0.00		
traT	Vr	38	0.18	0.39	0.00		

Variable	Group	N	Medias	D.E.	Medianas	H	p
fyuA	Vmt	11	0.45	0.52	0.00	5.06	0.0002
fyuA	Vq	48	0.02	0.14	0.00		
fyuA	Vr	38	0.13	0.34	0.00		

References

Vmt = MT GROUP

Vq = H GROUP

Vr = RB GROUP
