# Supplementary material for: Characterization of native Escherichia coli populations from bovine vagina of healthy heifers and cows with postpartum uterine disease
Source: PLoS One. 2020 Jun 1;15(6):e0228294. doi: 10.1371/journal.pone.0228294 (PMC7263596; doi:10.1371/journal.pone.0228294)
Supplement: S3 File — (DOCX) [file pone.0228294.s012.docx]

T test for independent samples (Infostat)

**Variable: Type 1 fimbriae - Clasific: Group - TEST: Bilateral**

Group 1 Group 2

H MT

n 48 11

Media 0.40 0.45

Variance 0.24 0.27

Media(1)-Media(2) - 0.06

LI(95) -0.39

LS(95) 0.28

pHomVar 0.7403

T -0.35

degrees of freedom 57

p-value 0.7263

Group 1 Group 2

H RB

n 48 38

Media 0.40 0.32

Variance 0.24 0.22

Media(1)-Media(2) - 0.08

LI(95) -0.13

LS(95) 0.29

pHomVar 0.7690

T 0.76

degrees of freedom 84

p-value 0.4486

Group 1 Group 2

MT RB

n 11 38

Media 0.45 0.32

Variance 0.27 0.22

Media(1)-Media(2) - 0.14

LI(95) -0.19

LS(95) 0.47

pHomVar 0.6108

T 0.84

degrees of freedom 47

p-value 0.4051

**Variable:** **Curli/cellulose -Clasific: Group - TEST:Bilateral**

Group 1 Group 2

H MT

n 48 11

Media 0.60 0.64

Variance 0.24 0.25

Media(1)-Media(2) - -0.03

LI(95) -0.36

LS(95) 0.30

pHomVar 0.8488

T -0.19

degrees of freedom 57

p-value 0.8467

Group 1 Group 2

H RB

n 48 38

Media 0.60 0.79

Variance 0.24 0.17

Media(1)-Media(2) - -0.19

LI(95) -0.38

LS(95) 0.01

pHomVar 0.2617

T -1.85

degrees of freedom 84

p-value 0.0672

Group 1 Group 2

MT RB

n 11 38

Media 0.64 0.79

Variance 0.25 0.17

Media(1)-Media(2) - - 0.15

LI(95) -0.45

LS(95) 0.15

pHomVar 0.3634

T - 1.03

degrees of freedom 47

p-value 0.3083

**Variable: Motility - Clasific: Group - TEST: Bilateral**

Group 1 Group 2

H MT

n 48 11

Media 0.52 0.91

Variance 0.25 0.09

Media(1)-Media(2) - -0.39

LI(95) -0.71

LS(95) -0.07

pHomVar 0.0820

T -2.44

degrees of freedom 57

p-value 0.0177

Group 1 Group 2

H RB

n 48 38

Media 0.52 0.34

Variance 0.25 0.23

Media(1)-Media(2) - 0.18

LI(95) -0.03

LS(95) 0.39

pHomVar 0.7646

T 1.66

degrees of freedom 84

p-value 0.0997

Group 1 Group 2

MT RB

n 11 38

Media 0.91 0.34

Variance 0.09 0.23

Media(1)-Media(2) - 0.57

LI(95) 0.26

LS(95) 0.88

pHomVar 0.1173

T 3.69

degrees of freedom 47

p-value 0.0006

**Variable: Biofilm - Clasific: Group - TEST: Bilateral**

Group 1 Group 2

H MT

n 48 11

Media 0.60 0.91

Variance 0.24 0.09

Media(1)-Media(2) - -0.30

LI(95) -0.62

LS(95) 0.01

pHomVar 0.0947

T -1.96

degrees of freedom 57

p-value 0.0500

Group 1 Group 2

H RB

n 48 38

Media 0.60 0.84

Variance 0.24 0.14

Media(1)-Media(2) - -0.24

LI(95) -0.43

LS(95) -0.05

pHomVar 0.0697

T -2.47

degrees of freedom 84

p-value 0.0155

Group 1 Group 2

MT RB

n 11 38

Media 0.91 0.84

Variance 0.09 0.14

Media(1)-Media(2) - 0.07

LI(95) -0.18

LS(95) 0.31

pHomVar 0.05039

T 0.55

degrees of freedom 47

p-value 0.5854
